# Evaluation of the Efficacy of Fish Skin Grafts as Wound Dressings: A Systematic Review

**DOI:** 10.3390/ebj6030050

**Published:** 2025-09-08

**Authors:** Jocelyn Ivana, I Gusti Putu Hendra Sanjaya

**Affiliations:** 1 Faculty of Medicine, Udayana University, Bali 80234, Indonesia; 2 Division of Plastic, Reconstructive and Aesthetic Surgery, Faculty of Medicine, Udayana University, Prof. Dr. I.G.N.G. Ngoerah General Hospital, Bali 80113, Indonesia

**Keywords:** fish skin graft, skin graft, xenograft, wound, wound dressing, burn dressing

## Abstract

The use of fish skin grafts as xenografts is a promising alternative for wound healing. Several studies have shown fish skin grafts to be a safer and more effective option compared to other alternatives, due to the large amount of fatty acids, including omega-3, which have been proven to promote wound healing. The purpose of this study was to evaluate the efficacy of fish skin grafts as wound dressing. A literature search up to March 2024 was conducted using the electronic databases of PubMed, Cochrane, and ScienceDirect. A total of 158 patients from six studies were included in this systematic review. All studies showed early wound healing using fish skin grafts; one study showed that wound healing was halved compared to paraffin gauze. Complete wound healing using fish skin grafts was noted as early as 30 days. Out of 114 patients treated with fish skin grafts, 1 patient showed signs of infection, and no patients showed allergic reactions. One study also found that fish skin grafts provide satisfactory wound scar quality. This study concludes that fish skin grafts are a great alternative and should be considered in wound treatment. The high omega-3 component that is preserved in fish skin grafts promotes faster wound healing and contains antibacterial agents that prevent infection. However, randomized control trials with a larger sample size are recommended to further assess the efficacy of fish skin grafts.

## 1. Introduction

Skin grafts are commonly used by physicians to promote healing and skin closure in nonhealing wounds, such as chronic and burn wounds [1,2]. There are several types of grafts that can be chosen, such as autografts (skin grafts obtained from the patient themselves), allografts (obtained from human donors), and xenografts (obtained from other species) [1]. Autografts are the standard treatment for definitive chronic wound closure due to their permanent skin integration, immunocompatibility, and their ability to retain the inherent structure of the skin [3]. However, large and extensive wounds might become a problem for autografts, as the patient probably does not have enough available skin to harvest [2,4].

Allografts and xenografts are the options for skin grafting to large and extensive wounds. Both grafts are commonly used as a temporary wound coverage while awaiting more definitive skin closure treatment or for patients needing resuscitation before autografting. Although allografts have the ability to undergo revascularization, they are used temporarily due to immune rejection and infection [3]. Allografts are more difficult to acquire and need to go through a long process before being able to be grafted to the patient to reduce the risk of contamination and transmitted disease [2]. Xenografts do not have the ability to revascularize, thus making them a temporary wound closure to promote healing. They are used in the short term as they will not integrate with the recipient’s tissue [3]. Bovine and porcine grafts are commonly used xenografts, although the use of fish grafts is rising due to the latest research that has shown them to be more beneficial. An ideal xenograft should be considered based on the strength, flexibility, adhesion to the wound, aesthetic result, and optimal wound healing. The safety of the graft should also be taken into consideration, such as the risk of disease transmission and rejection reactions. Bovine and porcine grafts need to go through a lengthy preservation process before being used to lower the risk of disease transmission. This lengthy process also often causes a large portion of collagen that could help with wound healing to be lost [2,4]. Meanwhile, fish skin grafts (FSGs) do not need to go through a lengthy preservation process, as they are considered to pose a lower risk of disease transmission compared to mammalian grafts [1,2]. An in vivo and in vitro study found that FSG collagen contents possessed good blood compatibility, had a higher cell viability than naturally derived biomaterials (T-1), and had a significantly lower immunological reaction compared to bovine tendons [5]. Consistent with other types of xenografts, FSGs serve as a temporary wound dressing. Other wound dressings that are commonly used are collagen alginate dressings and silver sulfadiazine cream 1%. Both dressings require a lot of dressing changes, thus resulting in more high-maintenance wound care [6].

Fish skin grafts may be harvested from Atlantic Cod or Nile Tilapia; both species live in different areas of the world, making FSGs more accessible worldwide. The preservation process of FSGs makes it possible to store them for up to 3 years at room temperature [2]. The FSG is found to be more porous compared to dehydrated amnion/chorion membrane allograft (dHACM); fish skin contains 16.7 holes per 100 µm on average, whereas the dHACM contains 1.7 holes per 100 µm on average. The average diameter of the pores in the fish skin graft is 16.1 µm, and that of the pores in the dHACM is 1.3 µm [7]. This allows more fibroblasts and keratinocytes to pass through, thus resulting in faster wound healing. Fish skins are rich in collagen (type 1 and 2), which are known to help in the third stage of wound healing. They also contain different fatty acids, which aid wound healing by affecting the production and activity of cytokines and competing with the inflammatory arachidonic acid to inhibit proinflammatory eicosanoid production. Among those fatty acids, omega-3 polyunsaturated fatty acid plays a huge role, including as an antibacterial agent that lasts for 48–72 h [2,8]. This is shown in a cytotoxicity assay, where it was found to have antimicrobial and anti-biofilm activity in vitro against *S. aureus*, *S. epidermidis*, and *P. aeruginosa*, as well as against multidrug-resistant *S. aureus* [9]. Omega-3 fatty acid also promotes wound healing by helping tissue regeneration by supporting revascularization and growth in the third and fourth stages of the wound healing process. FSG applications are relatively simple; FSG only needs to be soaked in 0.9% saline solution before being ready to be grafted on the patient. It can also be meshed, and only needs stitches or a medical patch to be applied [1]. The application of fish skin grafts has been seen in different cases in recent years, namely chronic diabetic foot ulcers, burns, chronic wounds, and even hidradenitis suppurativa [1].

Given the potential of fish skin grafts, we conducted a systematic review to assess the efficacy of fish skin grafts as wound dressings. The aim of this systematic review was to assess how fish skin grafts perform in the healing process of chronic wounds, mainly evaluating the duration of wound healing. Other factors that might impact the outcome of wound healing, such as infection and allergic reactions, will also be mentioned if available in the articles included in this study.

## 2. Materials and Methods

### 2.1. Search Strategy

**Figure 1:**
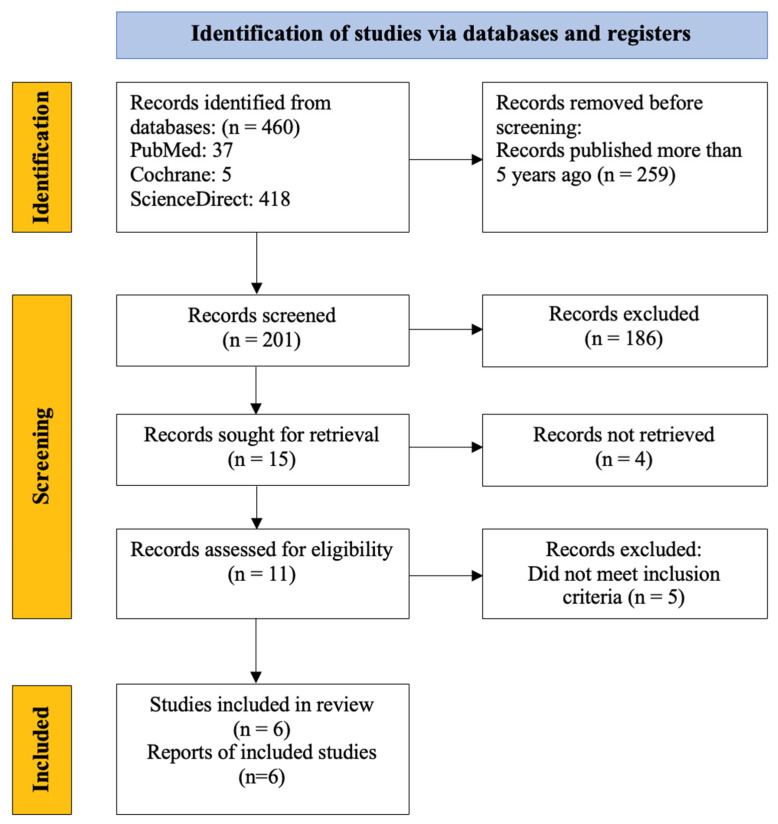
PRISMA flow diagram of the study search and identification process.

This systematic review was conducted in line with the “Preferred Reporting Items for Systematic Reviews and Meta-Analyses” (PRISMA) guideline. PubMed, Cochrane, and ScienceDirect databases were explored in March 2024 using “fish skin graft AND wound graft AND acellular fish skin” as keywords, resulting a total of 460 results. Any type of research that used fish skin grafts as a treatment option in humans, was written in English, had full text available, and was published in the past 5 years was included in this study. Figure 1 shows the PRISMA flow diagram indicating the study search and identification strategies.

### 2.2. Inclusion Criteria

This systematic review included original studies, such as randomized controlled trials, cohort studies, case series, and case reports, that met the other inclusion criteria. The other inclusion criteria were as follows: the research was published no more than five years ago, was performed on human subjects, and used fish skin grafts as a wound dressing, and the research objective was to find the efficacy of fish skin grafts.

### 2.3. Data Collection Process

The required data that were extracted from studies included in this research were independently extracted from every study by the authors using a standard data extraction format. Any identified discrepancies were resolved through discussion and consultation with experts. The data collected in each study were author(s), year of publication, study region, research design, sample characteristics, sample size, intervention, and outcomes.

### 2.4. Risk of Bias Assessment

A risk of bias assessment was conducted of all studies included in this systematic review using NIH study quality assessment tools. The quality assessment tools were used and adapted accordingly to accommodate all types of studies included in this systematic review. A total of 9 possible sources of bias were identified: research question (was the study question or objective clearly stated?); study population (were eligibility/selection criteria for the study prespecified and clearly described?); study representation (were the participants in the study representative of those who would be eligible for the intervention in the general or clinical population of interest?); participation (were all eligible participants that met the prespecified entry criteria enrolled?); sample size (was the sample size sufficiently large to provide confidence in findings?); intervention (was the intervention clearly described and delivered consistently across the study population?); outcome (were the outcome measures prespecified, clearly defined, valid, reliable, and assessed consistently across all study participants?); follow-up (was the loss to follow-up after baseline 20% or less, and were those lost to follow-up accounted for in the analysis?); and control of cofounders (were key potential confounding variables measured and statistically adjusted for their impact on the outcomes?).

Table 1 reports the summary of risk of bias evaluation of each study, with Y indicating yes, N indicating No, and N/A indicating the evaluation was not able to be performed for the study.

## 3. Results

### 3.1. Study Selection

The search results were then filtered to the past five years, leaving 201 records to be screened. These records were screened first by title and abstracts, excluding those that did not fit the objective of this study. In total, 186 records were excluded after the first screening process, leaving 15 records to be sought for full-text retrieval, where 4 could not be retrieved. The remaining 11 records that were able to be retrieved were then assessed based on the inclusion criteria. This further excluded five records, leaving six records to be included in this systematic review. This process was performed as indicated before in Figure 1.

### 3.2. Characteristics of Included Studies

The characteristics of each study included in this systematic review are displayed in Table 2. It details the year of publication, main author of the study, study type, sample size, test performed, and the result of each study.

In a cohort study performed by Badois N et al. [10], 21 patients were included and divided into two groups. The study was conducted on patients with head or neck cancer that went through surgery and needed a split-thickness skin graft (STSG) to cover free flap donor sites on the inner side of the ipsilateral arm. The size of the donor sites ranged from 30 to 45 cm^2^. The control group, which consisted of 10 patients, was treated using paraffin gauze, applied immediately after surgery, taken care of daily, and given a thick layer of Vaseline if the gauze had dried up. The dressing was then removed after four days and then changed every two days until complete re-epithelization. The second group consisted of 11 patients who were being treated with an acellular fish skin matrix soaked in saline solution immediately after surgery, then covered with moist compresses and semi-permeable dressing, and left in place until disintegrated. A new matrix was applied if re-epithelization was not completed by the time of matrix disintegration, up to three matrixes. The average healing time was 67.9 days in the control group and 31.5 days in the group treated with the acellular fish skin matrix. Overall, 60% (*n* = 6) of patients in the control group showed signs of local infection, while no patient in the second group showed any sign of local infection. Pain level decreased significantly in the second group; within 5 days after surgery, no patient had VAS of ≥3, versus 40% (*n* = 4) of patients in the control group who had VAS of ≥3. This study concluded that the use of the acellular fish skin matrix showed a decrease in local healing time, local infection, and pain level compared to standard wound care using paraffin gauze [10].

Lantis et al. [11] conducted a randomized controlled clinical trial on a total of 77 patients with chronic and unresponsive diabetic foot ulcers. The patients included had diabetic foot ulcers at least through the dermis but not extending to the tendon, muscle, or bone within the previous 4 weeks to 1 year. The treatment phase lasted for 12 weeks, and the patients were divided into two groups to evaluate wound closure after treatment. The first group consisted of 43 patients who were treated with fish skin grafts, secured with surgical adhesive strips, sutures, or staples. The second group of 34 patients was treated with collagen alginate therapy, changed once weekly by a medical professional, and changed twice or thrice weekly by the patient themselves. After 12 weeks, 56.9% (*n* = 29) of wounds treated with FSG healed completely, compared to 31.4% (*n* = 16) of wounds healed in the group treated with collagen alginate therapy. This study also calculated the cost-effectiveness of both therapies and found that FSG was more cost-effective compared to collagen alginate therapy [11].

Five pediatric patients were treated with FSG in a study by Cherry et al. [12] at the University Hospital of Lausanne, Belgium. Two patients received FSG due to a third-degree burn, one patient due to tinea capitis with fungal kerion that left a scalp defect, one patient due to scar excision of restrictive scar contracture post-thermal burn, and one due to axillary excision of hidradenitis suppurativa. The follow-up process ranged from 10 to 18 months, with all patients’ outcomes being satisfactory, showing no retraction, no hypertrophic scars, and complete wound coverage. Complete epithelialization took 29–62 days (mean = 48.6 days), and shrinkage in the wound surface after a few days was followed by early wound granulation. No hypersensitivity or allergic reaction was reported in this study [12].

Reda et al. [13] performed a study to evaluate the use of FSG in burn and blast injuries in armed conflict areas. Three cases were presented in the study; all had one initial debridement and simple wet-to-dry dressing, and then a fish skin graft was applied alongside negative pressure wound therapy. Wounds were then followed up after 7 days; all showed early wound granulation and no infection. FSGs were found to be an ideal alternative to allografts that need an ultra-low temperature for storage. Minimal training and no complex equipment are needed to apply FSGs, making them ideal for use in austere environments [13].

In a study conducted by Biazar et al. [14], acellular fish skin was used alongside growth factor to treat a 6 × 4 cm deep-thickness wound on the anteromedial surface of the distal tibia. After 30 days, the wound size reduced to 3 × 2 cm, and the wound had closed completely after 60 days. This study concluded that the fish skin graft was found to reduce wound healing time, with better scar quality [14].

A retrospective study performed by Michael et al. [15] evaluated the efficacy of FSG in the healing of full-thickness diabetic foot ulcers. In total, 51 patients with a total of 58 diabetic foot ulcers were included in this study, with a mean wound area of 3.02 cm^2^. After 16 weeks of treatment with an acellular fish skin graft, wound area was reduced by 87.57% from its initial size, and 60.34% (35 out of 58) of the wounds had healed completely [15].

## 4. Discussion

Acellular fish skin as a xenograft promises a lot of benefits towards chronic wound healing, namely burns and diabetic foot ulcers. Other known xenografts are harvested from mammals, posing the risk of disease transmission, thus requiring harsh processing before being able to be grafted. Harsh processing removes components that may help in wound healing, such as lipids, glycans, elastin, hyaluronic acids, and soluble collagens [1]. Out of the six studies that were reviewed, all studies show that FSG accelerates wound healing. Although all studies showed that FSG is efficacious towards wound healing, these studies were performed on different samples and varied demographics, one of which is the wound type that was treated. Lantis et al. [11] and Michael et al. [15] both treated diabetic foot ulcers, where Lantis found that the percentage of wounds healed after 12 weeks was increased in patients treated with FSG (56.9%) compared to those treated with collagen alginate therapy (31.4%). Michael et al. showed complete wound healing and wound size reduction of 87.57% from its initial size after 16 weeks [15]. Reda et al. found early wound granulation in their study that was performed on full-thickness blast and burn injuries; this was found on the first follow-up, 7 days after grafting [13]. Badois et al. concluded that healing time using FSG (mean = 31.5 days) was halved, compared to paraffin gauze (mean = 67.9 days). This study was conducted on a thin skin donor site, comparable to an intermediate or deep second-degree burn [10]. The age groups were also varied between the studies; studies performed by Biazar et al. [14] and Cherry et al. [12] were carried out in pediatric patients, unlike other studies reviewed. Biazar et al. found complete wound healing in their study after 60 days on deep-thickness wounds treated with FSG [14]. According to Cherry et al., complete re-epithelization using a fish skin graft took 29–62 days, with early wound granulation found only after a few days [12]. Wound areas were halved after 30 days, according to Biazar et al. [14]. Despite the variation in study designs, FSG shows promising, consistent results in promoting wound healing across different wound types and age groups.

These findings may be explained by the biological properties of fish skin grafts and the retention of structural and biochemical components such as type I collagen and omega-3 fatty acids after processing. Fiakos et al. analyzed FSG properties that may help in wound healing. It was found that fish skin has a similar structure to human skin, with both composed of three basic layers: epidermal, intermediate, and basal epithelial layers [16]. This study also mentioned the porous structure of FSG, which allows ingrowth and attachment of human fibroblasts. It also highlights the high omega-3 content of FSG (19% of its overall lipid content) compared to mammalian grafts (0.5% of its lipid content) [16]. While these components are hypothesized to support wound healing, the direct causal relationship between omega-3 content and improved clinical outcomes remains to be fully established. Seth et al. [17] discussed the potential role of omega-3 fatty acids—specifically eicosapentaenoic acid (EPA) and docosahexaenoic acid (DHA)—in modulating inflammation, enhancing the skin barrier, and providing antimicrobial effects. These fatty acids have been shown to influence the production of inflammatory cytokines such as IL-1β and IL-6. However, these mechanistic insights are primarily based on experimental or preclinical data, and further clinical studies are needed to confirm their contribution to wound healing when delivered via FSG.

Reports of adverse events associated with FSG are limited. Four studies—by Badois et al. [10], Cherry et al. [12], Reda et al. [13], and Biazar et al. [14]—explicitly noted that no infections or allergic reactions occurred in patients treated with FSG, although these findings were not supported by detailed safety monitoring protocols or definitions. Lantis et al. [11] reported one infection in the FSG group, compared to five in the collagen alginate control group. While these observations suggest a favorable safety profile, the absence of systematic adverse event reporting across studies limits the strength of this conclusion. The potential antibacterial and anti-biofilm properties of omega-3 fatty acids such as eicosapentaenoic acid (EPA) and docosahexaenoic acid (DHA)—components retained in FSG—may contribute to infection control. These fatty acids have demonstrated in vitro efficacy against pathogens including Staphylococcus aureus (both MRSA and non-resistant strains), Vibrio vulnificus, and Candida albicans [17]. However, the clinical relevance of these mechanisms in the context of FSG use remains to be validated in controlled trials. Several studies reported positive outcomes regarding scarring, pain, and cost when using fish skin grafts (FSGs), though the extent of quantitative assessment varies.

Cherry et al. [12] noted satisfactory scar appearance in pediatric patients treated with FSG, including the absence of retraction and hypertrophic scars. However, the authors acknowledged the study’s reliance on subjective outcome measures and called for the use of standardized tools for pain and scar assessment to enhance data robustness. In contrast, Biazar et al. [14] reported patient-reported pain scores between 1 and 4 out of 10, indicating a generally low pain experience, though no validated pain scale was used. Badois et al. [10] applied the Visual Analog Scale (VAS) and observed a significant reduction in pain (VAS ≥ 3 at five days; *p* = 0.0034). Lantis et al. [11] was the only study to perform a cost-effectiveness analysis comparing fish skin grafts (FSGs) with collagen alginate therapy (CAT). While the cost per healed ulcer was higher for FSG than CAT in per-protocol analysis (USD 7364 vs. USD 3989), the annualized cost—accounting for ongoing treatment of nonhealing ulcers—favored FSG. Based on Medicare data and modeled outcomes, the total annual cost per patient was USD 13,926 for FSG compared to USD 16,744 for CAT, indicating a USD 2818 cost saving with FSG. These findings suggest that FSG may contribute to improved patient comfort and reduced treatment cost. However, more rigorous studies using validated pain assessment tools and structured cost-effectiveness evaluations are needed to support these observations.

There were several limitations in this study. Most studies reviewed in this study have a small sample size; therefore, studies with larger, well-designed randomized controlled trials with standardized outcome measures still need to be carried out. Another limitation was that there were not a lot of studies published that compared the efficacy of FSG and other wound dressings, especially the other grafts available. Further research around the use of fish skin grafts still needs to be explored. Despite these limitations, the available evidence suggests that FSG is a promising option for wound healing, particularly in complex or chronic wounds.

## 5. Conclusions

This study found that acellular fish skin grafts (FSGs) are a promising option for wound healing, particularly in chronic and complex wounds such as burns and diabetic foot ulcers. Across diverse patient populations and wound types, FSG has demonstrated consistent benefits in accelerating healing, reducing pain, and improving scar quality. Its favorable safety profile and the retention of bioactive components—such as type I collagen and omega-3 fatty acids—further support its therapeutic potential.

However, the strength of this evidence is limited by small sample sizes, lack of standardized outcome measures, and limited direct comparisons with other grafts or advanced wound dressings. Reports of adverse events and limited cost-effectiveness are inconsistent and often anecdotal. Therefore, large-scale, randomized controlled trials with rigorous clinical and safety evaluation are essential to establish the comparative effectiveness of FSG and guide its broader adoption in clinical practice.

## Figures and Tables

**Table 1:** Summary of risk of bias evaluation of each study.

	Badois N. et al. [10]	Lantis J.C. et al. [11]	Cherry I. et al. [12]	Reda F. et al. [13]	Biazar E. et al. [14]	Michael S. et al. [15]
Research question	Y	Y	Y	Y	Y	Y
Study population	Y	Y	Y	Y	N/A	Y
Study representation	Y	Y	Y	Y	Y	Y
Participation	Y	Y	Y	N	N/A	N
Sample size	Y	Y	Y	N	N	Y
Intervention	Y	Y	Y	Y	Y	Y
Outcome	Y	Y	Y	Y	Y	Y
Follow-up	Y	N	Y	Y	N/A	Y
Control of cofounders	N	Y	N	N	N	Y

**Table 2:** Characteristics of each study included in this systematic review.

Author	Year	Study Type	Sample Size	Test Performed	Result
Badois N. et al. [10]	2019	Prospective, comparative, before–after cohort study	21	Radial forearm free flap donor site covered with either acellular fish skin or paraffin gauze	Healing time using acellular fish skin was halved; pain level and local infection rate were reduced
Lantis J.C. et al. [11]	2023	Prospective, multicenter, randomized controlled trial	77	Chronic diabetic foot ulcer treated with either fish skin graft or collagen alginate therapy	Fish skin graft healed significantly more wounds and reduced annual treatment cost
Cherry I. et al. [12]	2023	Retrospective, case series	5	Fish skin graft used in five different pediatric patients	Fish skin graft accelerated healing and improved post-operative pain control
Reda F. et al. [13]	2023	Case series	3	Large-area full-thickness burn and blast injuries treated with fish skin graft	Fish skin graft achieved early granulation tissue formation and lower infection risk
Biazar E. et al. [14]	2023	Case report	1	Deep thickness wound treated with fish skin graft	Reduction in wound healing time; better functional and aesthetic outcome
Michael S. et al. [15]	2019	Retrospective study	51	Diabetic foot ulcer treated with acellular fish skin	Acellular fish skin promoted rapid wound healing and rapid wound surface area reduction in the first 4 weeks

## Data Availability

No new data were created in this study.

## Abbreviations

The following abbreviations are used in this manuscript:

FSG	Fish Skin Graft
VAS	Visual Analogue Scale
EPA	Eicosapentaenoic Acid
DHA	Docosahexaeoic Acid
CAT	Collagen Alginate Therapy
